# Targeted next generation sequencing identified a high frequency genetic mutated profile in wood smoke exposure-related lung adenocarcinoma patients

**DOI:** 10.18632/oncotarget.25369

**Published:** 2018-07-17

**Authors:** Giovanny Soca-Chafre, Norma Hernández-Pedro, Alejandro Aviles-Salas, Carmen Alaez Versón, Karol Carrillo Sánchez, Andrés F. Cardona, Federico Avila-Moreno, Pedro Barrios-Bernal, Diana Flores-Estrada, Oscar Arrieta

**Affiliations:** ^1^ Personalized Medicine Laboratory, Instituto Nacional de Cancerología (INCAN) México City, México; ^2^ Department of Pathology, INCAN, México City, México; ^3^ Translational Genomics Laboratory, Instituto Nacional de Medicina Genómica (INMEGEN), México City, México; ^4^ Clinical and Translational Oncology Group, Institute of Oncology, Clínica del Country, Bogotá, Colombia; ^5^ Universidad Nacional Autónoma de México (UNAM), Facultad de Estudios Superiores (FES) Iztacala, Biomedicine Research Unit, Cancer Epigenomics and Lung Diseases Laboratory 12, México State, México; ^6^ National Institute of Respiratory Diseases (INER) “Ismael Cosío Villegas”, Research Unit, México City, México; ^7^ Thoracic Oncology Clinic, INCAN, México City, México

**Keywords:** lung adenocarcinoma, wood smoke exposure, genotyping, mutation profile

## Abstract

**Background:**

Wood smoke exposure (WSE) has been associated with an increased risk of lung cancer development. WSE has been related with high frequency of EGFR mutations and low frequency of KRAS mutations. The aim of this study was to evaluate large scale genomic alterations in lung adenocarcinomas associated with WSE using targeted next generation sequencing.

**Methods:**

DNA multi-targeted sequencing was performed in 42 fresh-frozen samples of advanced lung adenocarcinomas. The TruSeQ Cancer Panel (Illumina) was used for genomic library construction and sequencing assays.

**Results:**

WSE rate was higher in women (p=0.037) and non-smokers (p=0.001). WSE correlated with mutations in the genes SMARCB1 (p=0.002), Ataxia telangiectasia mutated (p=0.004), Kinase Insert Domain Receptor (p=0.006), and were borderline significant in RET and EGFR exon. Genomic alterations significantly co-occurred in the tumor suppressor gene ATM with the following genes: SMARCB1, EGFR exon 7, RET and KDR. Clinical factors associated with poor prognosis were ECOG ≥ 2 (p= 0.014), mutations in KDR (p= 0.004) and APC genes (p < 0.001).

**Conclusions:**

Lung adenocarcinoma patients with WSE showed a distinctive mutated profile for the SMARCB1, ATM, EGFR exon 7, RET and KDR genes. ECOG status and KDR gene mutations were significantly associated with poor prognosis.

## INTRODUCTION

Lung cancer is the first cause of cancer-related deaths worldwide with 1.6 million deaths per year [1]. In México, lung cancer accounts for 10% of all cancer-related mortality [2]. The recurrent etiological factor of non-small cell lung cancer (NSCLC) is cigarette smoking, represented by almost 90 % of patients in United States [3]. In México only 56.5% of NSCLC cases have a history of tobacco smoking, particularly, in women represents only 33% [4–6]. This suggest that other environmental factors have a greater impact in the development of lung cancer, such as asbestos exposure, arsenic, hydrocarbons, metals, ionizing radiation, air pollution, tuberculosis and wood smoke exposure (WSE) [7, 8].

Currently about 3 billion people, particularly females use biomass and coal as fuels indoors and for domestic cooking exposing themselves to WSE [9]. WSE in women is considered a risk factor for lung cancer independently of smoking status [10]. Wood combustion releases polycyclic aromatic hydrocarbons such as naphthalene, retene, and phenanthrene. *in vitro*, these carcinogens cause DNA strand breaks, epithelial-mesenchymal transition, cell proliferation and inflammation [11] and induce lung adenocarcinoma in mice [12].

Our group has previously reported that WSE is related with 35% of NSCLC cases in México [5]. Patients with WSE have showed a better response to treatment with tyrosine kinase inhibitors (TKI) targeting EGFR mutations [7]. Furthermore, it has been described that patients with WSE were associated with adenocarcinoma histology and higher incidence of EGFR mutations in up to 50% of the cases and low frequency of KRAS mutations with 6.7% [8]. Moreover, we reported gene expression profile of WSE-related NSCLC where 37 genes were significantly altered and closely related to UBC and GABARAPL1 affecting PI3K/AKT and MAPK pathways [13]. WSE is related to high levels of phosphorylated TP53, as well as promoter methylation in genes such as p16 and GATA4 [14]. However, a comprehensive genetic mutation profile in WSE-NSCLC patients and their clinical outcomes remains unexplored. The aim of the present work was to study somatic mutations based on genomic profiling by the method of targeted next generation sequencing (NGS) on tumor samples of lung adenocarcinoma patients with WSE and their prognostic value.

## RESULTS

**Figure 1:**
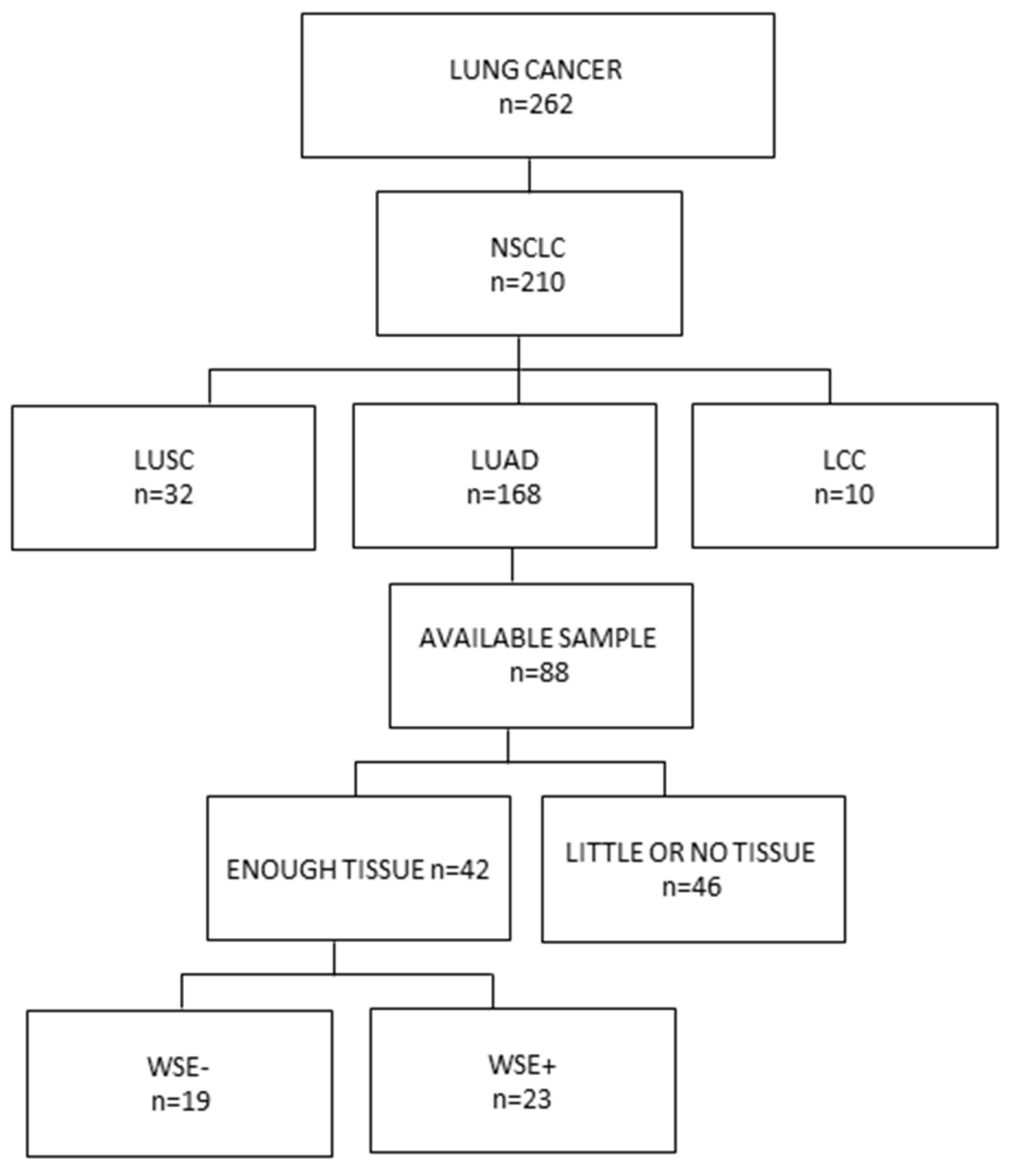
Consort diagram of patients included in the study From 262 patients with lung cancer 210 were classified as non-small cell lung cancers including 32 lung squamous cell carcinomas, 10 large-cell lung carcinomas and 168 lung adenocarcinomas. Samples were available for 88 cases and 42 with sufficient tissue were processed for DNA extraction, library construction and massive parallel sequencing. NSCLC: Non-small cell lung cancer, LUSC: lung squamous cell carcinoma, LUAD: lung adenocarcinoma, LCC: Large-cell lung carcinoma, WSE: Wood smoke exposure.

Patient selection for this study is outlined in Figure 1. From the patients with lung adenocarcinoma 71.4% were women. Median age was 67 years, 69% of the patients were over 60 years old and 85.7% had an ECOG of 0-1. Forty-five percent had a history of WSE and 38.1% had tobacco smoking history, but only 12.5% (2 cases) had both exposures (Table 1).

**Table 1:** Clinical characteristics according to WSE in NSCLC patients

		ALL (N=42)	WSE (-)	WSE (+)	p-Value
			(N=23)	(N=19)	
		% (n/N)	% (n/N)	% (n/N)	
**Gender**	Female	71.4(30/42)	43.3 (13/30)	56.7 (17/30)	
	Male	28.6 (12/42)	83.3 (10/12)	16.7 (2/12)	**0.037**
**Age**	Median (Range)	67(36-82)	67(36-82)	66(37-77)	0.552
	< 60 years	31 (13/42)	53.8 (7/13)	46.2 (6/13)	
	≥ 60 years	69.0 (29/42)	55.2 (16/29)	44.8 (13/29)	1
**WSE Index**	WSE index Median (Range)	72 (64.4-249.6)	NA	72 (64.4-249.6)	
**Tobacco-Smoking Exposure**	Smoking index Median (Range)	0 (0-55)	3 (0-55)	0 (0-0)	**< 0.001**
	Non-smoker	61.9 (26/42)	34.6 (9/26)	65.4 (17/26)	
	Smoker	38.1 (16/42)	87.5 (14/16)	12.5 (2/16)	**0.001**
**ECOG PS**	0-1	85.7 (36/42)	58.3 (21/36)	41.7 (15/36)	
	2+	14.3 (6/42)	33.3 (2/6)	66.7 (4/6)	0.384
**Predominant histological pattern**	Lepidic	14.3 (6/42)	66.7 (4/6)	33.3 (2/6)	0.878
	Acinar	33.3 (14/42)	50.0 (7/14)	50 (7/14)	
	Papillary	7.1 (3/42)	66.7 (2/3)	33.3 (1/3)	
	Solid	45.2 (19/42)	52.6 (10/19)	47.4 (9/19)	
**Histological Grade**	Low	45.2 (19/42)	52.6 (10/19)	47.4 (9/19)	0.818
	Intermediate	40.5 (17/42)	52.9 (9/17))	47.1 (8/17)	
	High	14.3 (6/42)	66.7 (4/6)	33.3 (2/6)	
**Disease Stage**	IIIB	21.4 (9/42)	77.8 (7/9)	22.2 (2/9)	
	IV	78.6 (33/42))	48.5 (16/33)	51.5 (17/33)	0.149
**CNS Metastases**	Absent	69.7 (23/33)	52.2 (12/23)	47.8 (11/23)	
	Present	30.3 (10/33)	40 (4/10)	60 (6/10)	0.708
**Lung Metastases**	Absent	72.7 (24/33)	45.8 (11/24)	54.2 (13/24)	
	Present	27.3 (9/33)	55.6 (5/9)	44.4 (4/9)	0.708
**Liver Metastases**	Absent	90.9 (30/33)	46.7 (14/30)	53.3 (16/30)	
	Present	9.1 (3/33)	66.7 (2/3)	33.3 (1/3)	0.601
**Bone metastases**	Absent	72.7 (24/33)	54.2 (13/24)	45.8 (11/24)	
	Present	27.3 (9/33)	33.3 (3/9)	66.7 (6/9)	0.438
**CEA**	<10 ng/mL	41.5 (17/41)	52.9 (9/17)	47.1 (8/20)	
	≥10 ng/mL	58.5 (24/41)	54.2 (13/24)	45.8 (11/24)	1

Abbreviations: ECOG PS: Eastern Cooperative Oncology Group Performance Status; CNS: Central Nervous System, CEA: Carcinoembryonic antigen, NA non-applicable

Predominant histological subtypes were solid (45.2%) and acinar (33.3%). Most patients presented high (45.2%) or intermediate (40.5 %) tumor grade, according to the American Joint Committee on Cancer 2010, 21.4% was stage IIIB and 78.6% stage IV. Central nervous system (30.3%), lung and bones (27.3%) were the main metastatic sites. Carcinoembryonic antigen (CEA) levels were higher than ≥ 10 ng/ml in 58.5% of the patients.

### Genomic profiling

Mutations frequencies are presented in Table 2. Over 40% were EGFR variants (59.5%), most frequently in exon 7 (40%), concurrent mutations in exons 7 and 21 (24%), concurrent mutations in exons 7 and 19 (12%), as well as exon 19 microdeletions (12%), while the L858R point mutation in exon 21 and exons 2/3 were 4%. TP53 mutations represented 50%, SMARCB1 45.2%, and both ATM and FGFR 42.9%. A group of mutations with frequencies in the range of 20% - 40% were present in HNF1A (38.1%), RET (35.7%) and KDR (21.4%). Other mutations with less than 20% frequency were detected in VHL, ERBB4, MET, STK11, CTTNB1, APC, NOTCH1 and CSF1R genes.

**Table 2:** Association between genomic alterations and patients with WSE

		ALL (N=42) % (n/N)	WSE (-) (N=23) % (n/N)	WSE (+) (N=19) % (n/N)	*p*-Value
**EGFR**	Wild Type	40.5 (17/42)	64.7 (11/17)	35.3 (6/17)	
	Mutant	59.5 (25/42)	48 (12/25)	52.0 (13/25)	0.353
**EGFR Exon 7**	Wild Type	54.8 (23/42)	69.6 (16/23)	30.4 (7/23)	
	Mutant	45.2 (19/42)	36.8 (7/16)	63.2 (12/19)	**0.061**
**EGFR by exon**	Exon 7	40 (10/23)	40 (4/10)	60 (6/10)	
	Exon 7/21	24 (6/23)	33.3 (2/6)	66.7 (4/6)	
	Exon 7/19	12 (3/23)	33.3 (1/3)	66.7 (2/3)	
	Exon 19	12 (3/23)	66.7 (2/3)	33.1 (1/3)	
	Exon 21	4 (1/23)	100 (1/1)	0 (0/1)	
	Exon 2	4 (1/23)	100 (1/1)	0 (0/1)	
	Exon 3	4 (1/23)	100 (1/1)	0 (0/1)	0.583
**TP53**	Wild Type	50.0 (21/42)	47.6 (10/21)	52.4 (11/21)	
	Mutant	50.0 (21/42)	61.9 (13/21)	38.1 (8/21)	0.536
**SMARCB1**	Wild Type	54.8 (23/42)	78.3 (18/23)	21.7 (5/23)	
	Mutant	45.2 (19/42)	26.3 (5/19)	73.7 (14/19)	**0.002**
**ATM**	Wild Type	57.1 (24/42)	75 (18/24)	25.0 (6/24)	
	Mutant	42.9 (18/42)	27.8 (5/18)	72.2 (13/18)	**0.004**
**FGFR**	Wild Type	57.1 (24/42)	54.2 (13/24)	45.8 (11/24)	
	Mutant	42.9 (18/42)	55.6 (10/18)	44.4 (8/18)	1.000
**HNF1A**	Wild Type	61.9 (26/42)	57.7 (15/26)	42.3 (11/26)	
	Mutant	38.1 (16/42)	50 (8/16)	50 (8/16)	0.753
**RET**	Wild Type	64.3 (27/42)	66.7 (18/27)	33.3 (9/27)	
	Mutant	35.7 (15/42)	33.3 (5/15)	66.7 (10/15)	**0.055**
**KDR**	Wild Type	78.6 (33/42)	66.7 (22/33)	33.3 (11/33)	
	Mutant	21.4 (9/42)	11.1 (1/9)	88.9 (8/9)	**0.006**
**VHL**	Wild Type	81 (34/42)	58.8(20/34)	41.2 (14/34)	
	Mutant	19 (8/42)	37.5 (3/8)	62.5 (5/8)	0.433
**ERBB4**	Wild Type	81 (34/42)	58.8 (20/34)	41.2 (14/34)	
	Mutant	19 (8/42)	37.5 (3/8)	62.5 (5/8)	0.433
**MET**	Wild Type	88.1 (37/42)	54.1 (20/37)	45.9 (17/37)	
	Mutant	11.9 (5/42)	60 (3/5)	40 (2/5)	1.000
**STK11**	Wild Type	88.1 (37/42)	54.1 (20/37)	45.9 (17/37)	
	Mutant	11.9 (5/42)	60.0 (3/5)	40.0 (2/5)	1.000
**CTTNB1**	Wild Type	88.1 (37/42)	54.1 (20/37)	45.9 (17/37)	
	Mutant	11.9 (5/42)	60 (3/5)	40(2/5)	1.000
**APC**	Wild Type	88.1 (37/42)	51.4 (19/37)	48.6 (18/37)	
	Mutant	11.9 (5/42)	80 (4/5)	20 (1/5)	0.356
**NOTCH1**	Wild Type	90.5 (38/42)	57.9 (22/38)	42.1 (16/38)	
	Mutant	9.5 (4/42)	25.0 (1/4)	75.0 (3/4)	0.313
**CSF1R**	Wild Type	90.5 (38/42)	57.9 (22/38)	42.1 (16/38)	
	Mutant	9.5 (4/42)	25.0 (1/4)	75.0 (3/4)	0.313

Abbreviations: EGFR: Epidermal growth factor receptor (ErbB-1 or HER1) TP53: Tumor protein p53 SMARCB1: SWI/SNF Related, Matrix Associated, Actin Dependent Regulator of Chromatin, Subfamily B, Member 1, ATM: Ataxia telangiectasia mutated gene, FGFR: Fibroblast growth factor receptor, HNF1A: Hepatocyte nuclear factor 1-alpha, RET: rearranged during transfection-receptor tyrosine kinase KDR: Kinase Insert Domain Receptor or Vascular Endothelial Growth Factor Receptor 2 VHL: von Hippel-Lindau tumor suppressor, ERBB4: Human Epidermal Growth Factor Receptor 4, MET: mesenchymal-epithelial transition factor receptor tyrosine kinase gene or Hepatocyte Gr owth Factor Receptor, STK11: Serine/threonine kinase 11 or liver kinase B1 (LKB1), CTTNB1: Catenin Beta 1, APC: Adenomatous polyposis coli gene, NOTCH1: Translocation-Associated Notch Protein TAN-1, CSF1R: Colony Stimulating Factor 1 Receptor.

**Figure 2:**
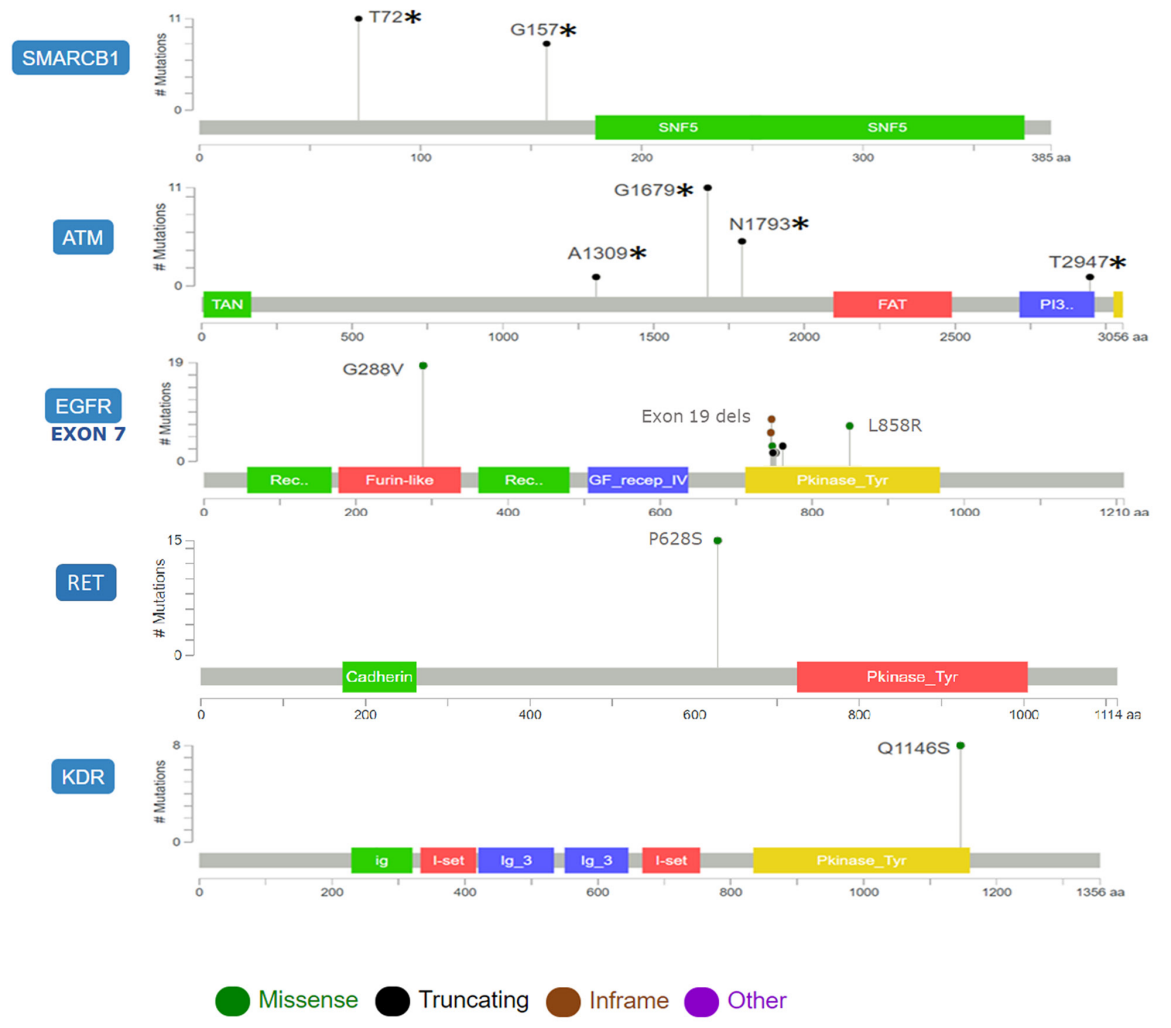
Diagram showing the distribution and types of mutation of genes associated with WSE in lung adenocarcinoma The position of lollipop markers indicates sites with mutations in different protein domains while the height is proportional to mutation frequency.

SMARCB1 mutations T72^*^ and G157^*^ were frameshifts upstream the conserved region of SNF5, the ATP-dependent nucleosome-remodeling complex that regulates transcription of multiple genes. Truncations A1309^*^, G1679^*^, N1793^*^, T2947^*^in ATM spanned the N-terminal domain containing motifs that interact with ATM substrates and c-Abl causing its activation following DNA damage, as well as the protein kinase domain. Missense mutations G288V in EGFR exon 7 affected the ligand binding domain (exons 5-7) in the extracellular portion of the receptor. RET and KDR showed missense mutations P628S and Q1146S near or at the protein tyrosine kinase domain respectively (Figure 2).

### Clinical and mutational features associated with WSE

Table 1 shows the clinical characteristics associated with WSE in all patients. WSE was associated with being female (p=0.037); non-smoker (p < 0.001), and lower tobacco smoking index (0 vs 3, p < 0.001). Five genes were more frequently mutated in WSE patients: KDR (89%, p=0.006), SMARCB1 (74%, p=0.002), ATM (72%, p=0.004), RET (67%, p= 0.055) and exon 7 of EGFR (63.2 %, p=0.061) (Table 2). A sub-analysis of the combination of five genes associated with WSE revealed that the majority of patients without WSE had no alterations in any of these five genes (65.2%). Conversely 57.9% of the cases showed alterations in four or the five genes in the presence of WSE (Table 3).

**Table 3:** Number of mutated genes according to WSE status

Wood-smoke exposure				Wood smoke exposure		
# expressed genes	Absent	Present		Absent	Present	Total
0	15	3	None	15	3	18
	**65.2%**	15.8%		**65.2%**	15.8%	42.9%
1	3	4	SMARCB1	2	3	5
	13.0%	21.1%		8.7%	**15.8%**	11.9%
			ATM	0	1	1
				0.0%	5.3%	2.4%
			EGFR7	1	0	1
				4.3%	0.0%	2.4%
2	0	0				
	0.0%	0.0%				
3	3	1	ATM+RET+EGFR7	2	0	2
	13.0%	5.3%		8.7%	0.0%	4.8%
			SMARCB1+ATM+EGFR7	0	1	1
				0.0%	5.3%	2.4%
			SMARCB1+ATM+RET	1	0	1
				4.3%	0.0%	2.4%
4	1	6	ATM+RET+KDR+EGFR7	0	1	1
	4.3%	31.6%		0.0%	5.3%	2.4%
			SMARCB1+ATM+KDR+EGFR7	0	1	1
				0.0%	5.3%	2.4%
			SMARCB1+ATM+RET+EGFR7	1	3	4
				4.3%	**15.8%**	9.5%
			SMARCB1+ATM+RET+KDR	0	1	1
				0.0%	5.3%	2.4%
5	1	5	SMARCB1+ATM+RET+KDR+EGFR7	1	5	6
	4.3%	**26.3%**		4.3%	**26.3%**	14.3%
						0

**Figure 3:**
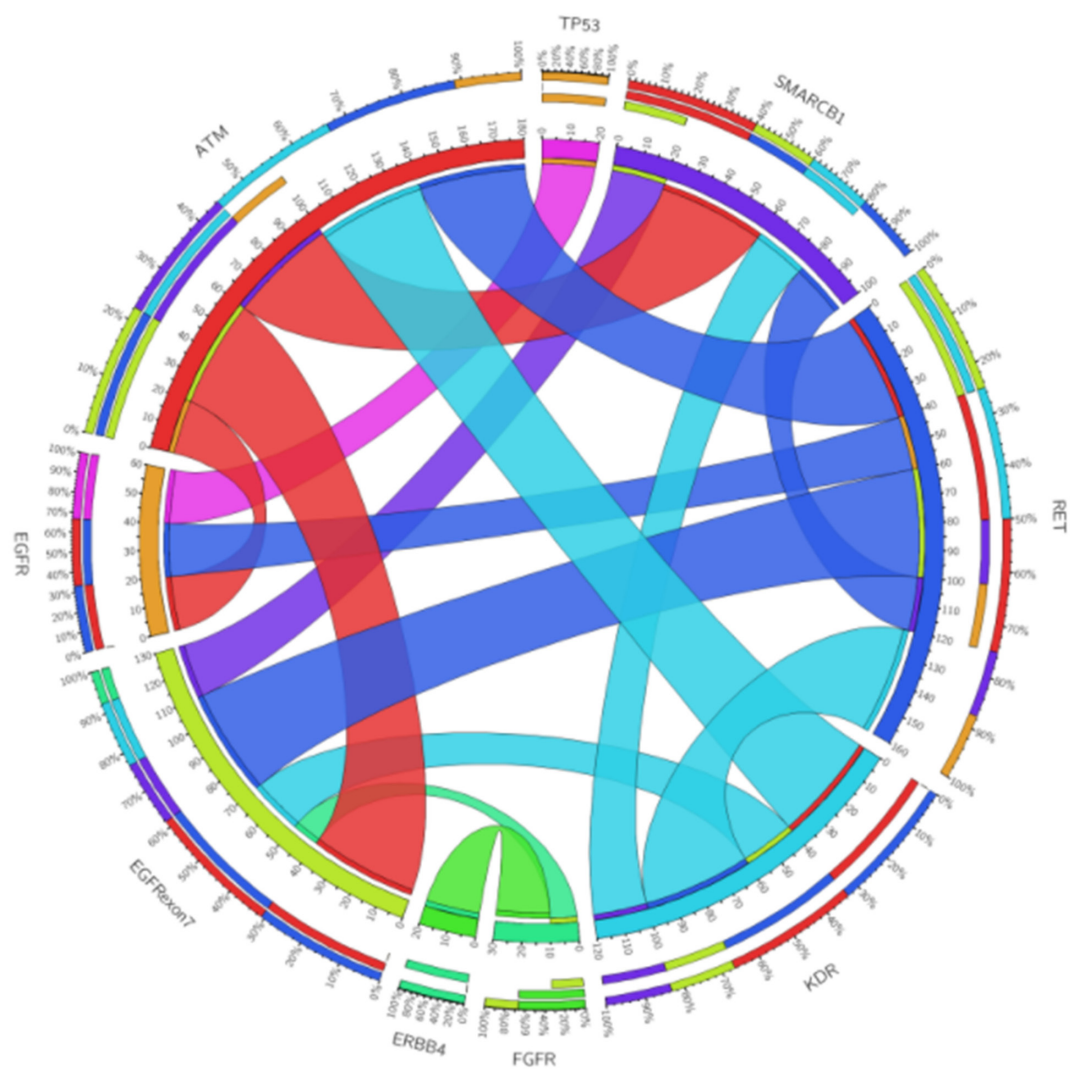
Circos plot of genomic alterations co-occurring in different genes The length of each arc represents the frequency of alterations in a particular gene that are related to other genes and the width of the ribbons connecting two genes represents the frequency of co-occurrence between them.

**Figure 4:**
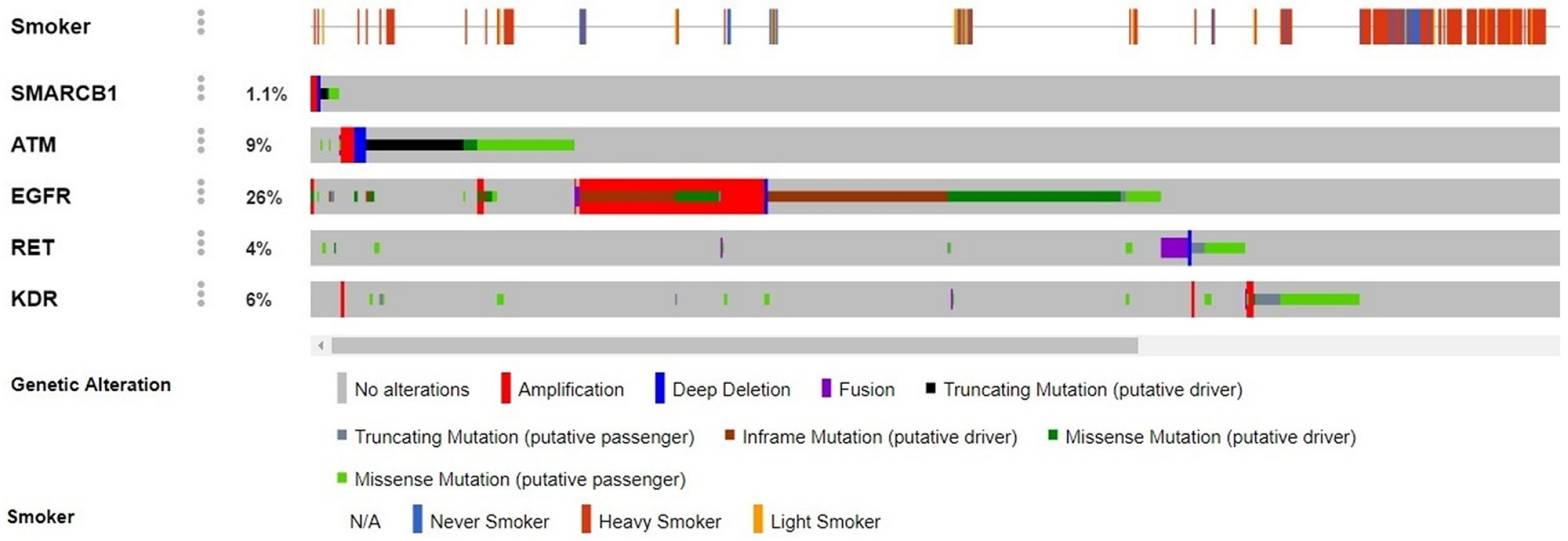
Contrast between genomic alterations in the genes SMARCB1, ATM, EGFR, RET and KDR vs. smoking history The frequencies of gene alterations were obtained from five comprehensive genomic studies of lung adenocarcinoma at the cBioPortal for Cancer Genomics (www.cbioportal.org): Broad Cell 2012, MSKCC 2015, TCGA Nature 2014, TCGA provisional and MSK-IMPACT with 1890 patients.

Concurrent mutations appeared mainly in the tumor suppressor gene ATM in the following combinations: ATM/RET, ATM/KDR (100% double mutants vs. 0% wild type), ATM/EGFR exon 7 (93.8% vs 6.2%) and ATM/SMARCB1 (73.7% vs 26.3%). Moreover, somatic mutations in oncogenes co-occurred mostly in RET/EGFR exon 7 (81.3% vs. 18.7%) and RET/KDR (88.9% vs. 11.1%), all of these mutations had statistically significant differences (p < 0.001) (Figure 3). Interestingly, these alterations were not associated with smoking history (Figure 4).

### Genetic and clinical factors that influence overall survival

**Figure 5:**
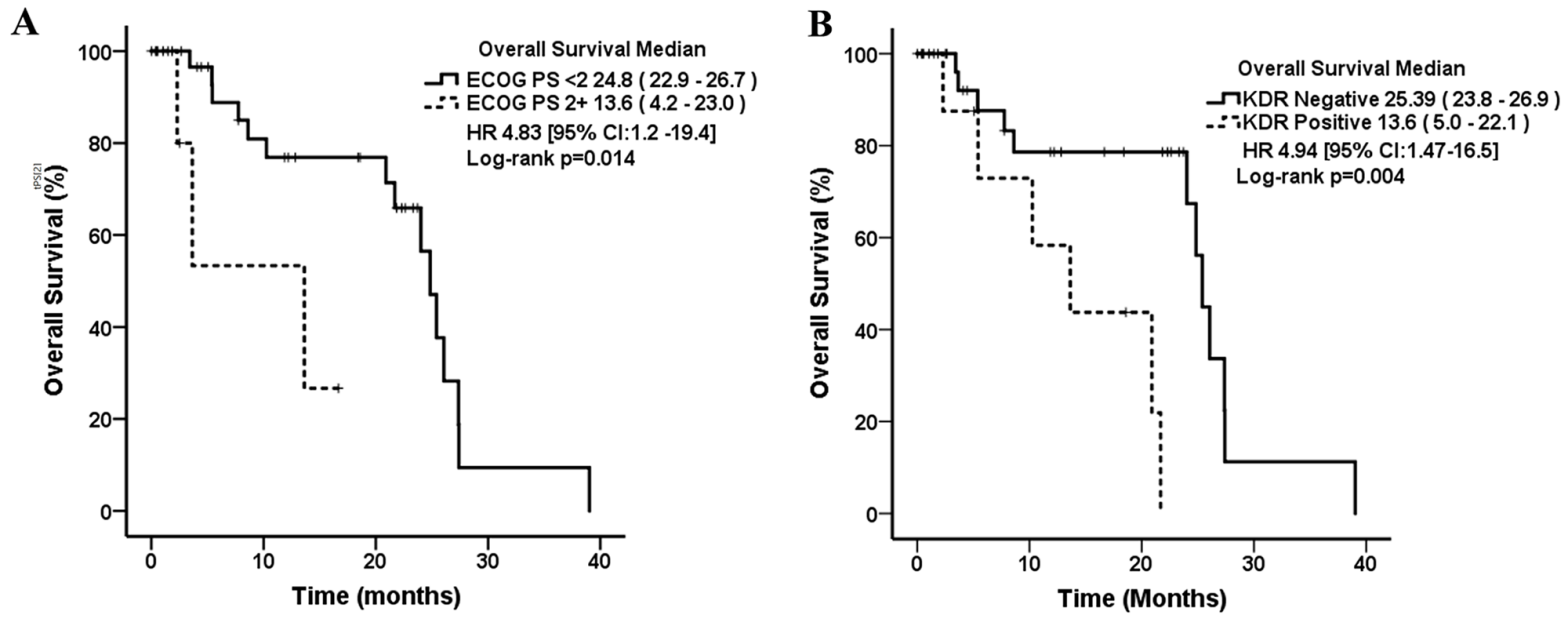
Kaplan–Meier curves for overall survival in lung adenocarcinoma patients associated with WSE according to their clinical and molecular characteristics **(A)** ECOG PS: Eastern Cooperative Oncology Group Performance Status. **(B)** KDR: Kinase Insert Domain Receptor or Vascular Endothelial Growth Factor Receptor 2 (VEGFR-2).

Median overall survival was 24.8 months (95% CI, 20.3 – 29.3). In the univariate analysis, patients with a higher ECOG performance status (2-3) had a median OS of 14 months compared to ECOG 0-1 with 25 months (p=0.014) (Table 4). Patients harboring KDR mutations were associated with poor prognosis (14 vs 25 months, p=0.004) (Figure 5).

**Table 4:** Univariate analysis of the factors associated with overall survival

		Mean, 95% CI	p-Value
OVERALL		24.8 (20.3-29.3)	
Gender	Female	24.8 (18.6-31.0)	
	Male	24.0 (NR)	0.367
Age	< 60 years	24.8 (0.0-51.9)	
	≥ 60 years	24.0 (19.8-28.2)	0.913
Tobacco exposure	Non-smoker	20.8 (8.8-32.9)	
	Smoker	24.8 (23.4-26.2)	0.457
WSE	Absent	24.8 (23.2-26.4)	
	Present	20.8 (10.9-30.8)	0.703
ECOG PS	0-1	24.8 (22.9-26.7)	
	2+	13.6 (4.2-23.0)	**0.014**
Disease Stage	IIIB	13.6 (0.0-50.6)	
	IV	3.0 (18.7-30.8)	0.864
Histological Grade	High	25.3 (NR)	
	Intermediate	24.0 (19.6-28.3)	
	Low	20.8 (4.8-36.9	0.524
EGFR status	Wild Type	24.0 (9.2-38.8)	
	Mutant	25.3 (18.4-32.3)	0.923
ATM	Wild Type	25.3 (24.2-26.5)	
	Mutant	21.6 (18.1-25.2)	0.660
KDR	Wild Type	25.3 (23.8-26.9)	
	Mutant	13.6 (5.0-22.1)	**0.004**
SMARCB1	Wild Type	25.3 (8.2-42.5)	
	Mutant	21.6 (18.1-25.2)	0.893
EGFR exon 7	Wild Type	25.3 (24.2-26.5)	
	Mutant	21.6 (18.3-25.0)	0.810
APC	Wild Type	24.8 (22.9-26.7)	
	Mutant	3.4 (1.7-5.0)	**<0.001**

Abbreviations: ECOG PS: Eastern Cooperative Oncology Group Performance Status, NR: Not reached, EGFR: Epidermal Growth Factor, ATM: Ataxia telangiectasia mutated gene, KDR: Kinase Insert Domain Receptor or Vascular Endothelial Growth Factor Receptor 2, SMARCB1: SWI/SNF Related, Matrix Associated, Actin Dependent Regulator Of Chromatin, Subfamily B, Member 1, EGFR exon 7: Epidermal Growth Factor exon 7, MET: mesenchymal-epithelial transition factor receptor tyrosine kinase gene or Hepatocyte Growth Factor Receptor, APC: Adenomatous polyposis coli gene.

## DISCUSSION

Around 40% of the world population uses solid fuels, including wood for cooking and heating homes. In Mexico, 15% of households, particularly in rural areas (40.5%) and with low socioeconomic status use wood as fuel for cooking. The development of chronic obstructive pulmonary disease in 62% of women is not attributable to tobacco, and could be related to long-term WSE. This is associated with the observed two-fold increase in lung cancer, particularly in nonsmoking Mexican women [15]. Previous reports have shown the association between WSE and lung cancer development mainly in women [7, 8, 13].

Exposure to carcinogenic compounds of wood smoke produce alterations in 53, phospho-TP53, and MDM2 expression increasing lung cancer risk [8]. Previously, our group reported a relation between WSE, female gender, EGFR mutations and different gene expression profiles [8, 13]. Our population is a complex admixture of races and ethnic groups and difficult to characterize therefore we do not make distinctions according to races in our study. This could be accomplished more accurately by genetic ancestry testing [16, 17]. On this study we describe a landscape of genomic alterations in lung adenocarcinoma patients with WSE in the tumor suppressors SMARCB1 and ATM, in addition to the oncogenes EGFR, RET and KDR.

Some of these genomic alterations are not reported in the catalog of somatic mutations in cancer (COSMIC), and may have a prognostic value for lung adenocarcinoma patients. Additionally, a comprehensive search across major genomic studies in lung adenocarcinoma revealed that these WSE-related genes are not associated with smoking history [18], showing a distinct mutation profile (Figure 4).

In the present study, we report mutations in known tumor suppressor genes such as SMARCB1 and ATM. Truncating SMARCB1 mutations were detected in 14 patients with a history of WSE and were indicative of poor prognosis. SMARCB1 is a member of the SWI/SNF chromatin remodeling complex involved in DNA repair and replication thereby controlling cell growth and differentiation [19]. Truncating forms of SMARCB1 are linked to an aggressive tumor phenotype, and are frequent in malignant rhabdoid tumors and epithelioid sarcomas, but rarely found in NSCLC [20]. Loss-of-function in the SWI/SNF complex activates EGFR-related pathways and represent a resistance mechanism to MET and ALK inhibitors, therefore, this could be a suitable target for combined inhibition with TKIs [21].

Our findings also report frequent frameshift mutations in the ATM tumor suppressor gene leading to protein truncation in patients with lung adenocarcinoma. This is consistent with the fact that ATM mutations represent an early event in NSCLC pathogenesis and over 40% of lung adenocarcinomas are negative for ATM protein expression [22]. This gene has been found to be deficient serving as an independent prognostic factor associated with worse survival in stages II/III and chemotherapy resistance [22]. Moreover, several ATM polymorphisms are risk factors for developing lung cancer in never smokers with low levels of carcinogen exposure [23]. Upon loss of ATM function, patients experience genomic instability that can be targeted through inhibition of alternative DNA repair mechanisms in combination with TKIs, which result in better response and overall survival [22, 23].

Furthermore, we report genomic alterations in the oncogenes EGFR, RET and KDR. The average frequency of EGFR mutations in Latin America is 30%, as we have describe it on behalf of the Latin American Consortium for the Investigation of Lung Cancer (CLICaP) in two comprehensive studies [24, 25]. Roughly 90% of these mutations are exon 19 deletions and the L858R mutation in exon 21. We have also reported the presence rare mutations in EGFR in exons 18-21 of the tyrosine kinase domain in 20.5% of the patients [25]. In the present study, we report novel mutations in exon 7 of EGFR encoding for an extracellular portion of this receptor. Alterations in this region could affect ligand binding and the activation of intracellular pathways as well as the response to antibody-based therapies such as cetuximab. [26].

ATM mutations are an early event in NSCLC pathogenesis mutually exclusive with TP53 mutations and may substitute its functional role in cancer initiation. Loss of ATM function contributes to genomic instability impairing double-strand break (DSB) DNA repair, therefore, combined treatments with inhibitors for alternative DNA repair mechanisms have been tested. ATM-deficient NSCLC cells reported higher sensibilization to ionizing radiation after cisplatin treatment and *in vivo* studies showed increased sensitivity to cisplatin and AZD6738 [27].

Furthermore, we describe the presence of missense mutations close to the tyrosine kinase domain of the RET oncogene. There is a 2.5% incidence of RET missense mutations in NSCLC. These mutations spanning the extracellular cadherin-like and the intracellular tyrosine kinase domains affect downstream signaling pathways promoting tumorigenesis [18, 20]. However, the most studied RET alterations in NSCLC are gene fusions mutually exclusive with EGFR mutations. NSCLC patients with RET rearrangements are generally young, never smokers, with high grade and small tumors of solid subtype. RET translocations are currently targeted with different TKIs but to date there are no therapies available for RET mutations.

In addition, we detected missense mutations in the tyrosine kinase domain of the KDR gene encoding the vascular endothelial growth factor receptor 2 (VEGFR-2) that were associated with shorter overall survival. The cBioportal database reveals a frequency of KDR mutations of 8% and 1% amplifications in NSCLC also the expression level of the VEGFR-2 protein defines molecular subsets of this malignancy. VEGFR-2 mediates the activation of EGFR-related pathways and its high expression is correlated with poor prognosis indicating a clinically attractive target with multiple VEGFR TKIs treatment [18]. However, responses to anti-VEGFR-2 antibodies or TKIs are still limited, with better response rates and PFS than conventional therapies but no significant improvements in OS [28].

Some of the genomic alterations detected in WSE-related NSCLC in our study were concurrent, represented mostly by ATM mutations in combination with another tumor suppressor, like SMARCB1 and oncogenes such as RET, KDR and EGFR exon 7 [29]. We hypothesize that carcinogens released by WSE produce frameshift truncations, resulting in loss of protein function in the tumor suppressors ATM and SMARCB1, and subsequent mutations in the oncogenes RET, KDR and EGFR exon 7 among others involved in the development of lung adenocarcinoma. The association between these three oncogenes may highlight the activation of several signaling pathways associated to tyrosine kinase receptors, suggesting the use of TKI combinations could be a suitable therapeutic strategy and would explain better response rates observed in NSCLC patients with WSE [8, 24]. Patients with driver alterations in major oncogenes, such as ALK, ROS1 and EGFR can benefit from targeted therapies, however, the presence of concurrent mutations in tumor suppressor genes can alter the course and prognosis of the disease [26, 27]. Our study is based on a small cohort, and due to the limited number of patients these results should be taken with caution since there is always a small probability of false positives, but this could be elucidated in further studies that focus on the role of these genes in NSCLC associated with WSE.

## MATERIALS AND METHODS

### Patient selection

A prospective cohort study was conducted, in patients diagnosed with lung adenocarcinoma from 2014-2017 at the Thoracic Oncology Clinic of the Instituto Nacional de Cancerología. The protocol was approved by the scientific and ethics institutional committees (15/049/ICI and CEI/1023/15, respectively). A total of 42 patients participated in the project after signing informed consent. A detailed medical history was registered including characteristics of patients, such as: age, gender, smoking status, WSE, disease stage, histological classification and clinical outcome. WSE was defined as exposure to fumes resulting from burning wood in fireplaces and wood stoves for at least four hours a day over five years. The WSE exposure index was calculated as the average number of hours spent cooking daily per total number of years, as reported previously by Behera [30].

### Sample processing

Tissue samples were obtained by tru-cut needle biopsies from primary tumors and they were immediately frozen in liquid nitrogen prior to storage until DNA extraction and library preparation. The pathology department performed the histologic diagnosis and quantification of the percentage of neoplastic cellularity. The procedure for DNA extraction and purification was carried out using the Genomic DNA Wizard kit (Promega, Madison, WI, USA). DNA purity was assessed by a NanoDrop-1000 spectrophotometer (Thermo Scientific, Wilmington, DE, USA), concentration was measured using a Quantus fluorimeter, and the DNA integrity was tested by agarose electrophoresis.

### Library preparation and sequencing

The commercial TruSeq Cancer Panel (llumina) for 48 cancer-related genes and 212 amplicons was used (FC-130-1008, Illumina; San Diego CA, USA). Targeted sequencing was performed on a MiSeq instrument, with an average sequencing depth per base of 1000X. ALK fusions were detected by Fluorescent *in Situ* Hybridization.

### Sequence analysis and variant calls

The bioinformatic workflow used for sequence analysis was the following: FASTQ files generated in the sequencer were processed in the FASTQC program. Sequences were filtered with the Trimmomatic software removing adapters. Those sequences with phred quality scores over Q30, *i.e* with base calling accuracy of 99.9% aligned with BWA using hg19 as reference genome. They were subsequently processed with the PICARD tools package, preparing the alignments for GATK analysis. Genomic sites with high propensity to insertions or deletions were realigned. The quality of reads and alignments was recalibrated and variants were called with muTect. Statistical filters were applied to the variants obtained to distinguish actual mutations from possible artifacts. All filtered variants were annotated regarding their possible functional consequence by snpEff and Variant Studio and the alignments and variants were visualized in the Integrative Genomics Viewer (Broad Institute, USA).

### Statistical tests

Continuous variables were summarized as arithmetic means with standard deviation, medians with interquartile ranges for descriptive analysis, while categorical variables were expressed as frequencies and percentages. Either Student’s t or Mann–Whitney U tests were used for two group comparisons, according to data distribution evaluated by Kolmogorov-Smirnov test. Comparisons between categorical variables were assessed by Fisher’s exact or χ2 tests. A p-value < 0.05 was accepted as statistically significant for two tailed tests. All variables were dichotomized for survival curve analysis. Overall survival (OS) was measured from day of diagnosis to the date of death or last follow-up, and comparisons among survival times were performed with log-rank test. Data were analyzed using SPSS software package, version 22 (SPSS, Inc., Chicago, IL, USA).

## CONCLUSIONS

WSE-related lung adenocarcinoma presents genomic alterations in SMARCB1, ATM, EGFR exon 7, RET and KDR not associated with smoking history. Genomic changes in some of these genes had a relevant impact on overall survival in lung adenocarcinoma patients and could represent novel therapeutic targets. Further studies are required to elucidate the functional role of these genomic alterations in early events of WSE-related carcinogenesis and the implications of loss of function mutations in these tumor suppressor genes.

## Abbreviations

AKT: AKT serine/threonine kinase 1
ALK: Anaplastic lymphoma kinase
APC: Adenomatous polyposis coli gene
ATM: Ataxia telangiectasia mutated gene
c-Abl: Abelson murine leukemia viral oncogene homolog 1
CCDC6: Coiled-Coil Domain Containing 6
CEA: Carcinoembryonic antigen
COSMIC: Catalog of somatic mutations in cancer
CSF1R: Colony stimulating factor 1 receptor
CTTNB1: Catenin Beta 1
CUX1: Cut Like Homeobox 1
DCR: Disease control rate
DNA: Deoxyribonucleic acid
ECOG: Eastern Cooperative Oncology Group
EGFR: Epidermal Growth Factor Receptor
ERBB4: Epidermal growth factor receptor 4
ERK: Extracellular signal–regulated kinase
FGFR: Fibroblast growth factor receptor
FRMD4A: FERM Domain Containing 4A
GABARAPL1: Gamma-aminobutyric acid receptor-associated protein-like 1
GATA4: GATA binding protein 4
HNF1A: Hepatocyte nuclear factor 1-alpha
KDR: Kinase Insert Domain Receptor
KIAA1217: Sickle tail protein homolog
KIAA1468: LisH Domain And HEAT Repeat-Containing Protein KIAA1468
KIF5B: Kinesin Family Member 5B
KRAS: Kirsten rat sarcoma viral oncogene homolog
MAPK: Mitogen-activated protein kinases
MDM2: Mouse double minute 2 homolog
MET: Mesenchymal Epithelial Transition gene
MTOR: Mechanistic Target Of Rapamycin Kinase
MYC: MYC proto-oncogene
NCOA: Nuclear Receptor Coactivator 5
NGS: Next generation sequencing
NOTCH1: Neurogenic Locus Notch Homolog Protein 1
NSCLC: Non-small cell lung cancer
OS: Overall survival
P16: Cyclin-Dependent Kinase Inhibitor 2A (CDKN2A)
PFS: Progression-free survival
PI3K: Phosphoinositide-3-kinase
RET: Rearranged during transfection gene
ROS1: ROS Proto-Oncogene 1, Receptor Tyrosine Kinase
SMARCB1: SWI/SNF Related, Matrix Associated, Actin Dependent Regulator Of Chromatin, Subfamily B, Member 1
SNF5: SWI/SNF chromatin-remodeling complex subunit SNF5
STK11: Serine/Threonine Kinase 11
SWI/SNF: SWItch/Sucrose Non Fermentable family
TKI: Tyrosine kinase inhibitors
TP53: Tumor protein p53
TRIMM33: Tripartite motif-containing 33
UBC: Ubiquitin C gene
VEGFR-2: Vascular endothelial growth factor receptor 2
VHL: Von Hippel-Lindau tumor suppressor
WSE: Wood smoke exposure
